# Catastrophic health expenditures arising from out-of-pocket payments: Evidence from South African income and expenditure surveys

**DOI:** 10.1371/journal.pone.0237217

**Published:** 2020-08-11

**Authors:** Steven F. Koch, Naomi Setshegetso

**Affiliations:** 1 Department of Economics, University of Pretoria, Pretoria, Gauteng Province, Republic of South Africa; 2 Department of Economics, University of Botswana, Gaborone, South-East District, Republic of Botswana; Tulane University, UNITED STATES

## Abstract

This study examines catastrophic health expenditures and the potential for such payments to impoverish South African households. The analysis applies three different catastrophic expenditure measurements, and we apply them across four South African Income and Expenditure Surveys. Since households have limited resources, they are also limited in their capacity to purchase health care. Thus, if a household devotes a large share of that capacity to health care, it may not be able to cover other necessary expenses, which could be catastrophic. The measurements differ in their definition of household capacity. Despite the differences in measurements, and, therefore, results, we find limited incidence of health care expenditure catastrophe, although larger shares of capacity are being devoted to health care in more recent years. In line with the finding that catastrophe is rare, we find that very few households are subsequently impoverished, because of health care costs.

## Introduction

‘… in all countries, fairness in financial contribution embraces two critical aspects: that of risk pooling between the healthy and the sick and risk sharing across wealth and income levels.’ [1]

Prior to 1994, the South African government, through apartheid, developed political, land and economic policies that structured society according to population group, gender and age-based hierachies [2]. These restrictions greatly influenced the organisation of social life, access to basic amenities, such as: health care, labour markets and education. In health care, such discrimination meant that different population groups had their own health care departments [3]. The African majority were forced to reside in rural areas, where health systems were underfunded, while high quality care was skewed towards the health facilities serving the white minority in urban areas [4]. However, when the new government took power in 1994, considerable effort was invested in addressing the inherited inequities from the apartheid government.

In health care, one important reform was user fee abolition in government clinics in 1994 among the elderly, children below the age of six, and pregnant and nursing mothers (as long as they were not covered by a medical aid scheme) [2]. In 1996, the user fee abolition policy was further extended to the entire population, provided that they were not living in a household earning more than R100 000 per year in 1995 prices [5, 6]. Subsequent evidence suggests that “free” health care policies improved the utilisation of preventive care [7, 8], although curative care did not increase all that much [5, 9, 10]. Although much care was free, it appears that government expenditures on health were poorly targeted [11]. More recent research does suggest that poorer South African households are more likely to use the public sector, which provides indirect evidence that the poor are benefitting from user fee abolition [12, 13].

In some countries the removal of financial barriers has been ineffective in protecting households from the costs associated with adverse health events, due primarily to inadequate supplies of drugs and medical services, as well as poorer quality services at these facilities [14–17]. Since user fee abolition was supposed to lead to free prescription medicine for those eligible, similar issues may have arisen in South Africa [13, 18]. In many countries, the exemption schedule is not well understood [19]. South Africa’s proposal allowed for free coverage, as well as partially free coverage, although some users were also expected to pay the full amount [20]. Thus, it is possible that abolition did not have much of an impact on out-of-pocket health care payments, and, therefore, an analysis of financial protection in the health care system over time can help us understand whether financial protection deserves further policy emphasis.

The literature on financial protection, which relates to health care financing, often focuses on catastrophic health care expenditure. That literature covers a number of countries [21–24]. Some research has examined both health care financing and health care utilisation [25]. Analysing financial protection through CHE follows one of three methods [1, 26, 27]. In general, the literature does not compare results across methods or even over time, which limits what one can learn. For that reason, we do both. We examine financial protection through the potential for catastrophic expenditure and impoverishment. We do so applying multiple methods, allowing us to compare the differences in the level of financial protection that arise from the choice of method. We also extend the comparison across time to allow us to indirectly examine the gradient in financial protection over time.

## Methods

This PhD research was approved by the University of Pretoria, Faculty of Economic and Management Science, Research Ethics Committee.

The focus of this analysis is the potential cost of out-of-pocket payments, which is measured in different ways throughout the literature. In particular, we examine the potential for catastrophic health expenditures, as well as impoverishment due to health expenditures. The potential for catastrophe is determined by the ratio of out-of-pocket payments to three different measure of the household’s capacity-to-pay (or ability to pay). Intuitively, the larger that ratio (or share), the less remains of the household’s budget for other purchases, some of which could be necessary for survival. Thus, if a large share of the household’s capacity-to-pay is devoted to health expenditures, those out-of-pocket health expenditures could be catastrophic. Another way to examine catastrophe is to consider whether or not households are impoverished, because of their health expenditures. Intuitively, the concern is that some households are pushed to consume less than they need, because they have had to pay for health care.

### Capacity-to-pay

For the determination of the aforementioned shares, it is necessary to define the household’s capacity-to-pay. We define that capacity in three ways. One is non-subsistence expenditure [1]. In addition, we examine two household equivalance-based capacties-to-pay, one using total household expenditure and another using total household nonfood expenditure [26, 27].

Each of the three methods require a measure of equivalence, which is an adjustement used to make amounts comparable across households. One common equivalence measure used in macroeconomics is per capita income; however, that comparison does not pay attention to the fact that some populations may have a larger or smaller proportion of working age adults, and, therefore, per capita measures are less than perfect. The same is true at the household level; households that do not need to support children or grandparents could be relatively better off. For that reason, a range of equivalence measures has been proposed in the literature to account for these differences in household age structure [28]. However, there is evidence that the exact measure of equivalence used in this type of analysis does not impact catastrophic health expenditure, as defined by the World Health Organization [1, 29]. In the method outlined by the World Health Organization, $h_{e_{i}}$ also represents the number of consumption equivalents in the household, and it is determined by estimates from a log-log model of total food expenditure on household size; the household size parameter is estimated to be 0.56 using data from 59 countries, including South Africa [30]. This value, because it is close to 0.5, suggests that rather than dividing by the number of household members (which gives per capita consumption), we should divide by the square root of the number of household members. For that reason, and to keep some consistency in approach, we use the same equivalence scale for all three methods; see (1)).

$$\begin{array}{c} h_{e_{i}}={\left(A_{i}+\alpha K_{i}\right)}^{\theta } \end{array}$$ (1)

In (1), $h_{e_{i}}$ represents the number of household equivalent adults, where *A*_*i*_ is the number of adults and *K*_*i*_ is the number of children in household *i*. The parameter *α* represents the cost of each child, while *θ* reflects household economies of scale. Thus, the equation reflects the idea that children do not require as many resources as adults, and that at least some consumption activity is consumed as a family, rather than separately by each household member. This adjustment method has been extensively employed using South African data in the measurement of poverty. In those studies, *α* = 0.5 and *θ* = 0.9 is commonly used; therefore, we use these same values [31, 32].

The two easiest capacity-to-pay measures to understand are total household expenditure (denoted by *x*_*i*_) per adult equivalent and total nonfood expenditure (denoted by *z*_*i*_) per adult equivalent; see Eq(2) [26, 27]. The former is based on the sum of all household expenditures divided by $h_{e_{i}}$ from (1), while the latter is based on the sum of all household expenditures, except those made on food. For this measure, food includes tobacco, alcohol and food eaten away from home. Again, this latter value is divided by $h_{e_{i}}$. Capacity-to-pay can also be measured by total household non-subsistence expenditure [1]. This method, generally referred to as the Xu or WHO method, because of the author and his association with World Health Organization at the time, requires a few additional analytical steps.

$$ctp_{wb}=x_{i}/h_{e_{i}}$$ (2)

$$ctp_{nf}=z_{i}/h_{e_{i}}$$ (3)

The WHO method calculates food expenditure (which we denote by *f*_*i*_ for each household) to include all purchases on food, except for food eaten away from home. Furthermore, within this method, food expenditure does not include alchol or tobacco purchases. Food expenditure is used to calculate the share of the household’s budget devoted to food $\left(w_{f_{i}}=f_{i}/x_{i}\right)$, the middle of the distribution of which is used to identify subsistence households. Specifically, $I_{i}\left(w_{f}^{45}\leq w_{f_{i}}\leq w_{f}^{55}\right)$ indicates whether household *i*’s food share $\left(w_{f_{i}}\right)$ lies between the 45^th^ and 55^th^ percentile of the food share distribution; $I_{i}=1$ denotes that it does.

For households whose food share lies in the middle of the food share distribution, a weighted average of their equivalence adjusted food expenditure $\left(f_{e_{i}}=f_{i}/h_{e_{i}}\right)$ is calculated; see (4), in which *ω*_*i*_ is the household survey weight or the inverse of the probability that the household was to be included in the sample, based on the survey design.

$$\begin{array}{c} \ell =\frac{\sum_{i:I_{i}=1}\omega_{i}f_{e_{i}}}{\sum_{i:I_{i}=1}\omega_{i}} \end{array}$$ (4)

That weighted average (*ℓ*) is referred to as the povertly line, and it is per adult equivalent. That measure is used to determine each household’s adjusted poverty line or its subsistence level of food expenditure (*s*_*i*_), which is the poverty line times the number of adult equivalents in the household $\left(s_{i}=\ell \times h_{e_{i}}\right)$. The capacity-to-pay is meant to capture non-subsistence expenditure; see Eq (5). However, for households who spend less on food than the subsistence level, we use their nonfood expenditure.

$$\begin{array}{c} ctp_{who}=\{\begin{array}{cc} x_{i}-s_{i} & \;\text{if}\;s_{i}\leq f_{i}\\ x_{i}-f_{i} & \;\text{otherwise}\; \end{array} \end{array}$$ (5)

### Health expenditure shares and potential catastrophe

Out-of-pocket health care payments are determined by all health costs that are paid directly by the household. It does not include payments made by third parties, such as health insurers. Thus, it would include user fees and co-payments, over-the-counter and prescription drugs, as well as doctor fees, hospital costs and related charges, as long as they were paid by the household and not reimbursed. These costs are summed within all households in the various surveys and they are referred to as out-of-pocket payments for the household (*oop*_*i*_). Given a household’s capacity-to-pay and its out-of-pocket health care payments, the share of the household’s capacity-to-pay devoted to such payments is simply the ratio.

$$\begin{array}{c} oopctp_{i}=oop_{i}/ctp_{i} \end{array}$$ (6)

Whether or not a household has been catastrophically affected by these payments depends on the size of that ratio. Given an arbitrary proportion, *κ*, a household is defined as potentially facing catastrophic health expenditures (CHE) if the share exceeds that arbitrary value. We define an indicator of catastrophe for each household, which depends on the value of *κ*, $C_{i}^{\kappa }=I_{i}\left(oopctp_{i}\geq \kappa \right)$. For analysis purposes, in addition to *κ* = 0.4, which has been used by other authors in existing empirical studies, we considered payments to be catastrophic if they are equal to or exceed *κ* = 0.05, *κ* = 0.10 and *κ* = 0.15 to document evidence on those thresholds, as well [33–36].

### Impoverishment

Another worry that arises is whether or not a household has been made poor, because they have had to pay for health. Intuitively, a household is impoverished if their health payments push them below subsistence. In other words, their total expenditure without health expenses exceeds their subsistence level, but once health expenses are included, that is no longer the case; see Eq (7), where $P_{i}$ denotes whether household *i* is impoverished; again I is an indicator function equal to one if the expression is true.

$$\begin{array}{c} P_{i}=I_{i}(x_{i}\geq s_{i}\;\text{and}\;x_{i}-oop_{i}<s_{i}) \end{array}$$ (7)

### Software

In terms of our analysis, to arrange the data, we use both STATA version 15.1, along with R and the tidyverse package in R [37, 38]. In order to estimate weighted means, variances and quantiles, we use the Hmisc package developed for R [39]. Finally, we put our tables together directly within R using xtable [40].

### Data

Income and expenditure data is the primary source of data for the analysis of out-of-pocket payments, because we need information on expenditures. Therefore, we rely on data obtained from four nationally representative, cross-sectional South African Income and Expenditure Surveys (SAIES) conducted in 1995, 2000, 2005-06 and 2010-11 [41–44]. Each survey followed a two-stage stratified design with sampling of primary sampling units in the first stage and sampling of dwelling units in the next stage. The 1991 population census underpinned the sample frame for the 1995 SAIES household survey; however, there were issues affecting that census and subsequently the 1995 SAIES [45]. The 1991 population census was conducted under the apartheid regime, when the former “independent states” of Transkei, Bophuthatswana, Venda and Ciskei (TBVC) were not part of South Africa; thus, they were not included in the census and their size had to be estimated. However, only two of the states (Bophuthatswana and Venda) had district level information to allow such an estimation, necessitating the exclusion of the states lacking that type of data. In certain parts of the country, particularly rural areas in the former “independent states”, maps of enumeration areas were absent and households in these districts were not listed. Hence, it was difficult to include them in the census. Also, no attempt was made to incorporate new informal settlements, which were springing up all over the country. Since a newer census was not yet available, these problems carried over to the sampling frame for the household surveys conducted in 1995 and 1996. To address these problems, StatsSA has since recalculated the household weights for the 1995 SAIES, using the 1996 population census. These weights were used in the analysis. Fortunately, later survey frames were not similarly compromised; regardless, sample household weights from those surveys were also used, as required for the calculation of the poverty line—recall Eq (4) and the discussion preceding it—as well as for our estimates of catastrophe and impoverishment.

The South African Income and Expenditure Surveys collect household income and consumption expenditures on all relevant items for the analysis. With regard to out-of-pocket payments, they are measured as expenditures that were incurred by the household as a whole. Although each survey collected information on household payments, there was a minor difference in method, due to a change in the goods classification systems; see below. In order to calculate adult equivalence, we also collected information on the number of children (aged 14 and under) and adults in the household for each survey.

In 1995 and 2000 the Standard Trade Classification was used to define expenditures including health payments. During the survey, households were expected to recall expenditures they had made in the previous 12 months or in the past month, in some cases. The health expenses are captured as purchases made for medicine (prescription and non-prescription), hospital and clinic services and expenses on therapeutic appliances. From 2005-06 and 2010-11, Statistics South Africa switched to the Classification of Individual Consumption According to Purpose (COICOP). Furthermore, in 2005-06, different households were surveyed at different points in time over a 12-month cycle. Households were given diaries to record their consumption expenditures, rather than providing recall information on expenditures from the past month or year [46]. In the 2005-06 and 2010-11 SAIES, the health payments are captured as medicines (with and without prescription), medical products (such as bandages and syringes) and therapeutic devices (including spectacles, hearing aids and braces), hospital expenses and outpatient services, which are classified into medical, dental and paramedical. In line with the concern that unpredicted health payments might be catastrophic, we excluded expected and predictable costs, such as contributions to a medical aid scheme (health insurance) from health payments.

Apart from out-of-pocket payments, it is also necessary to capture food expenditure (with and without alcohol, tobacco and meals purchased away from home) and total expenditure in order to calculate all three capacity-to-pay measures. The household consumption aggregate includes both monetary and non-monetary expenses, such as gifts given to households, which is captured in the surveys. Consumption expenditure was used, rather than income, because it takes into account household coping mechanisms when their income is low [27]. In the short run, a household is able to “smooth” its consumption; hence it is reasonable to assume that consumption will be more directly related to a household’s current living standards than income, which may only be received intermittently [47]. The remaining expenditure items were taken from the surveys as either totals or calculated as totals within COICOP groups, depending on the survey year. For the 2006–06 and 2010–11 surveys, which were conducted over time, the expdenditure values are deflated to the month of March (March 2006 and March 2011). All expenditure data is converted to monthly values, where necessary.

## Results

In this section, in addition to summarising the analysis data, we present our main set of results across a series of tables.

### Data summary

We summarise the primary variables needed for our analysis in Table 1. These variables include: monthly household consumption on food, both with (WB) and without (WHO) alcohol, tobacco and food away from home; out-of-pocket health payments; nonfood consumption; and the number of adults and children in the household for the calculation of equivalence. The data, not adjusted for inflation, suggest increases in expenditure over time. Adjusting for inflation is not necessary, since all of the analysis is conducted within years, and the analysis focuses on ratios. If both the numerator and the denominator of a ratio are adjuste by the same inflation factor, the inflation factor cancels out of the ratio, and, therefore, is not needed. We also see that household sizes have decreased over the time period, primarily because there are fewer adults in each household, on average.

**Table 1:** Household structure and monthly expenditure outlays.

	IES 1995	IES 2000
	(*N* = 26, 710)	(*N* = 22, 437)
Food consumption (WHO)	553.02 (545.49, 560.54)	596.87 (590.03, 603.72)
Food consumption (WB)	635.81 (627.36, 644.27)	671.05 (662.86, 679.24)
Non-food consumption	2,385.79 (2,313.53, 2,458.06)	2,107.60 (2,020.92, 2,194.28)
Out-of-pocket payments	26.52 (25.18, 27.86)	16.01 (14.97, 17.05)
Total consumption	3,021.61 (2,945.49, 3,097.73)	2,778.65 (2,688.53, 2,868.77)
Total adults	2.92 (2.90, 2.94)	2.72 (2.70, 2.75)
Total kids	1.42 (1.41, 1.44)	1.38 (1.36, 1.40)
	IES 2005–06	IES 2010–2011
	(*N* = 20, 994)	(*N* = 25, 119)
Food consumption (WHO)	603.60 (594.72, 612.48)	955.20 (942.72, 967.67)
Food consumption (WB)	722.74 (711.90, 733.58)	1,161.73 (1,146.55, 1,176.91)
Non-food consumption	3,151.82 (3,064.57, 3,239.08)	5,496.45 (5,376.75, 5,616.14)
Out-of-pocket payments	65.51 (58.84, 72.17)	88.90 (82.63, 95.18)
Total consumption	3,874.56 (3,781.46, 3,967.67)	6,658.17 (6,531.55, 6,784.80)
Total adults	2.68 (2.66, 2.71)	2.59 (2.57, 2.61)
Total kids	1.33 (1.31, 1.35)	1.16 (1.14, 1.17)

This table reports means and 95% confidence intervals for household level information from each of the South African Income and Expenditure Surveys used in the analysis. The WHO definition of food expenditure does not include alcohol, tobacco and food purchased away from home. The WB definition does include alcohol, tobacco and food purchased away from home, which explains their differences. Out-of-pocket payments are those related to health expenditure, but does not include health insurance premiums or costs covered by third-party payers. Table prepared with xtable package in R [40]. The code for the development of this data is located in the Appendix; see S1 File.

### Incidence of catastrophic health expenditures

In Table 2, the results for catastrophic health expenditure (CHE) are presented; they are evaluated at various thresholds of out-of-pocket health expenditures as a share of a household’s capacity to pay. Recall that we use three separate capacities; thus, we have three panels in the table. To quickly summarise, the incidence of CHE is fairly low, regardless of the threshold or method, although, as expected CHE is higher at lower thresholds. In all cases, there is a significant jump in the percentages between 2000 and 2005–06 that is worthy of deeper investigation, but is beyond the scope of this analysis.

**Table 2:** The distribution of catastrophic health expenditure over time.

Panel A	IES 1995	IES 2000	IES 2005	IES 2010
Catastrophic ≥ 5%	4.7212 (4.475, 4.967)	4.8898 (4.608, 5.172)	11.8211 (11.384,12.258)	9.4446 (9.083, 9.806)
Catastrophic ≥ 10%	1.4361 (1.298, 1.574)	1.4477 (1.292, 1.604)	3.5206 (3.271, 3.770)	3.1664 (2.950, 3.383)
Catastrophic ≥ 15%	0.7191 (0.621, 0.817)	0.7553 (0.642, 0.869)	1.5903 (1.421, 1.759)	1.2592 (1.121, 1.397)
Catastrophic ≥ 25%	0.1603 (0.114, 0.207)	0.2653 (0.198, 0.333)	0.3879 (0.304, 0.472)	0.3676 (0.293, 0.442)
Catastrophic ≥ 40%	0.0357 (0.014, 0.058)	0.0801 (0.043, 0.117)	0.0942 (0.053, 0.136)	0.0688 (0.036, 0.101)
Panel B	IES 1995	IES 2000	IES 2005	IES 2010
Catastrophic ≥ 5%	10.6837 (10.326,11.042)	10.5543 (10.153,10.956)	25.0609 (24.475,25.647)	22.2307 (21.716,22.745)
Catastrophic ≥ 10%	4.2454 (4.012, 4.479)	4.3678 (4.101, 4.635)	11.6129 (11.179,12.046)	9.9740 (9.603,10.345)
Catastrophic ≥ 15%	2.1786 (2.009, 2.348)	2.4961 (2.292, 2.700)	6.4753 (6.142, 6.808)	5.4897 (5.208, 5.771)
Catastrophic ≥ 25%	0.8406 (0.735, 0.946)	1.0898 (0.954, 1.226)	2.7329 (2.512, 2.953)	2.1735 (1.993, 2.354)
Catastrophic ≥ 40%	0.2678 (0.208, 0.328)	0.4698 (0.380, 0.559)	1.0431 (0.906, 1.181)	0.9082 (0.791, 1.026)
Panel C	IES 1995	IES 2000	IES 2005	IES 2010
Catastrophic ≥ 5%	17.3380 (16.899,17.777)	17.8189 (17.319,18.319)	34.0974 (33.456,34.739)	30.3796 (29.811,30.948)
Catastrophic ≥ 10%	8.9570 (8.626, 9.288)	8.9496 (8.576, 9.323)	19.0413 (18.510,19.572)	16.0269 (15.573,16.480)
Catastrophic ≥ 15%	5.3708 (5.109, 5.632)	5.2992 (5.006, 5.592)	11.8450 (11.408,12.282)	9.7365 (9.370,10.103)
Catastrophic ≥ 25%	2.3120 (2.138, 2.486)	2.5093 (2.305, 2.714)	5.6856 (5.372, 5.999)	4.7162 (4.454, 4.978)
Catastrophic ≥ 40%	0.9745 (0.861, 1.088)	1.1303 (0.992, 1.269)	2.4770 (2.267, 2.687)	1.9868 (1.814, 2.159)

The table reports, for each survey year, the percentage of the the population facing catastrophic health care expenditures. We use thresholds of 5%, 10%, 15%, 25% and 40% shares of health expenditure relative to the household’s capacity-to-pay as defined in (5)) along with 95% confidence intervals. Panel A focuses on the WHO-method, where capacity is defined as non-subsistence expenditure [1]. Panel B defines the household’s capacity-to-pay by adult equivalent total household expenditure [26]. Panel C defines the household’s capacity-to-pay by adult equivalent total nonfood expenditure [27]. Table prepared with the xtable package in R [40]. The code to develop these values is available in Appendix; see S2 File.

The incidence of CHE at the 5% threshhold is around 5% of the population using non-susbsistence expenditure as the capacity (Panel A) covering 1995 and 2000; however, when capacity is defined as adult equivalent expenditure (Panel B) or adult equivalent nonfood expenditure (Panel C), CHE is around 10.5% and 17.5%, respectively. From 2005 to 2010, those population percentages are approximately double. In 1995 and 2000, CHE is in the neighbourhood of 10% for non-subsistence expenditure (Panel A) and 22-25% for equivalent total consumption (Panel B) and 30-34% for equivalent non-food expenditure (Panel C).

When we consider 15% incidence, i.e., the percentage of the population that is subject to spending 15% of their budgets on health care, it is much lower, and statistically significantly so—there is no overlap in the 95% confidence intervals. The incidence ranges from 0.7% of the population (Panel A) to 11% of the population (Panel C). Again, the values before the survey collection methodology changes are approximately one-half of the post-change values. For non-subsistence expenditure (Panel A), incidence rises from 0.7% before to 1.2-1.5% afterwards. When capacity is defined as equivalent total expenditure, incidence rises from 2.2% to about 6% (Panel B), whereas in Panel C, the rise is from around 5% to between 10-12%.

Finally, if we focus on the most concerning threshold, 40% of the capacity-to-pay expended on health care out-of-pocket, we find approximately 0.05% before to 0.07-0.09% after the COICOP change (Panel A). In Panel B, where the capacity is defined as adult equivalent total expenditure, we see between one-quarter and one-half percent incidence in 1995 and 2000. In 2005–06 and 2010–11, incidence is closer to one percent. For adult equivalent nonfood expenditure (Panel C), incidence increases from approximate one percent pre-COICOP to approximately 2% post-COICOP.

### Household impoverishment

Given the rather small CHE percentages, regardless of approach, it is not surprising that few households are subsequently impoverished due to OOP. The percentage of impoverished households is presented in Table 3, and these, like the CHE values, are weighted to the population using inverse probability weights obtained from each survey. Impoverishment was no more than 0.5% of the population in 2005, but was generally less than one-third of a percent for all of the other years.

**Table 3:** Impoverishment arising from out-of-pocket health care payments.

	IES 1995	IES 2000	IES 2005	IES 2010
Impoverished Households	0.1599	0.3198	0.4502	0.3346
95% Confidence Interval	(0.114,0.206)	(0.252,0.388)	(0.360,0.541)	(0.263,0.406)

The table reports the percentage of households made poor (along with confindence intervals), because of out-of-pocket health care payments, covering four South African Income and Expenditure Surveys (IES). These households would have had consumption in excess of subsistence, except that health care payments pushed them below subsistence; see Eq (7). All values weighted to the population. Table prepared with xtable package in R [40]. The code to develop these figures is available in Appendix; see S2 File.

## Discussion

From a financial protection perspective, the low percentage of households potentially facing catastrophic health expenditures suggests a health care system that does not financially burden its citizens. Given “free” health care, as outlined above, these results are both to be expected and suggestive of a well-functioning health system. However, caution must be exercised when interpreting these results too optimistically, as it could be that other factors are at play. Importantly, health expenditure calculations do not consider foregone earnings or travel costs. In the case of travel costs, nearly 3/4 of those, who felt that public health care was unaffordable in South Africa, felt that was because of travel costs [13]. For poor South Africans, who may be working either informally or with limited access to sick or family leave to care for sick family members, seeking health care would have the additional cost of reducing earnings. Such losses might force individuals or family members to forego health care, itself. Therefore, it is likely that we underestimate the potential for catastrophe; unfortunately, there is very limited data available to consider this in any detail. However, there is evidence that households in 1995 who had experienced an illness did seek health care, and there is further evidence that the reduction in user fees that occurred in 1994 and 1996 had only a limited impact on facility usage [9, 10, 48]. Despite that, there are also concerns that South Africans do not believe they can afford to use the public health care system [13].

While the literature does not have a benchmark against which to compare CHE results for the years 2000 and 2010-11, our findings for the years 1995 and 2005–06 are consistent with those previously documented for those data sets [30, 49]. For 1995, earlier research finds 0.03 percent CHE incidence (underpinned by non-subsistence expenditure, so comparable to Panel A results in Table 2) at the 40 percent threshold [30]. For 2005–06, previous research finds a slighlty smaller 0.07 percent at the 40 percent threshold, again, compared to Panel A results in Table 2 [49]. At the 40 percent threshold of non-food expenditure (Panel C), South Africa’s incidence is also lower than other African countries, such as Ghana, which is reported to have a CHE incidence of 2.4 percent, Kenya at 11.4 percent and Swaziland with 2.4 percent [50–52].

The broader macroeconomic, primarily government finance, data reported by the WHO—see Table 4 suggests that government expenditure on health has increased from 2000 to 2005. It is a larger share of current health expenditure, such that private contributions are also smaller. Relatedly, the overall share of health in the government budget has risen. These rises coincide with a reduction in the amount of health care funded out-of-pocket, at least as a percentage of current healht expenditure. In other words, at the macro-level, it does appear that (a) government is taking on a larger burden of health care costs. In fact, even though government spending on health as a percent of GDP fluctuates over time, it has remained above 5 percent—an ideal value for a country to make progress towards the provision of universal health coverage [53]. Despite the macro-level evidence, our household survey data suggests that potential catastrophe has risen, albeit from a very low base, which suggests that government’s increased efforts have not kept pace with health care needs.

**Table 4:** Macroeconomic data on health financing.

	2000	2005	2010
Out-of-pocket payments per capita (USD)	33.5	43.7	45.9
Out-of-pocket payments as % of current health expenditure	15.1	12.4	8.5
Current health expenditure per capita (USD)	221.8	354.1	539.6
Current health expenditure as % of GDP	7.4	6.7	7.4
Domestic general government health expenditure as % of GDP	2.7	2.8	3.9
Domestic general government health expenditure per capita (USD)	81.6	145.4	284.8
Domestic general government health expenditure as % of current health expenditure	36.8	41.1	52.8
Domestic general government health expenditure as % of general government expenditure	10.9	9.8	12.4
Domestic private health expenditure as % of current health expenditure	61.7	57.6	44.2

The data in this table is sourced from the WHO Global Health Observatory Data Repository. Available at: https://apps.who.int/gho/data/node.main.GHEDOOPpUSSHA2011?lang=eng.

As noted, we uncovered very low levels of potential catastrophe. Given the rather small CHE percentages, it was not surprising that we uncovered few households that are impoverished due to out-of-pocket payments for health care. Compared to other developing and African countries, South Africa’s impoverishment is lower. In Mongolia, 12 percent of households are found to be impoverished by OOP payments, 7 percent in Swaziland, 18 percent in Uganda and about 9 percent in Ghana [50, 51, 54, 55].

Unfortunately, there are a few caveats to this analysis. As we discussed earlier, there are differences in pre- and post-COICOP Income and Expenditure data classifications and collection processes; thus, it is likely that the data is not entirely comparable. Previous research has looked at the comparability of some expenditure measures across the income and expenditure surveys of 1995, 2000 and 2005–06 picking out an array of differences [56]. In particular, pre-COICOP relied on recall, which is likely to miss small payments, while the post-COICOP diary method may miss large, especially out-of-pocket, values (since such payments are relatively rare). Despite those differences, it is the data that is available for this analysis.

It should also be noted that households may not understand health expenditures to be what analysts understand them to be. For example, households may not record expenses on traditional (but private) practitioners, such as spiritual healers and sangomas, as health expenditures. It is also possible that households will not completely capture health expenses for all household members. While it is not possible to address these problems, we acknowledge that these problems are bound to limit the degree to which some conclusions can be drawn. Taking all these problems and challenges into consideration, it is fortunate that health payments are defined similarly across all surveys, allowing the analysis undertaken in this study.

## Conclusion

This research put together data from four South African Income and Expenditure Surveys, covering the first 15 years of the republic post-Apartheid; we were not able to include a 2015-16 survey, because it did not take place. We used the data to extract information about primarily food, health care and total expenditures, which we used to examine the potential for catastrophic health expenditures, as well as the possibility that paying for health care led some households to become impoverished.

We described and employed three common approaches from the literature for examining catastrophe finding differences in the results they yielded. Despite that fact, the results confirm that South Africans do not generally face exhorbitant out-of-pocket expenses, when they receive health care. Given that the public health care system has limited fees, while the private system is generally used by those who have access to third-party payments, these results are not surprising.

However, the results also suggest that the change in approach adopted by Statistics South Africa to change their income and expenditure data collection methods may have improved the reporting of health care expenditures. Before 2005, the statistical agency collected expenditure data in the Spring (October in South Africa) following a recall method. In 2005–06, they switched to a diary method, such that much less recall was required. They also surveyed across the year and the country, incuding during the winter months; it is possible that the collection of data during cold and flu season might have led to increased coverage of over-the-counter medicines and increased overall reported out-of-pocket health care expenses. A deeper analsysis of response patterns across the different surveys and survey months, in the case of the 2005 and later surveys, might allow future research to develop adjustments for the data to improve comparability.

## Supporting information

S1 File R File for descriptive tables. This file provides the R code for developing the descriptive statistics tables.
(R) Click here for additional data file.

S2 File R File for catastrophic health expenditure and impoverishment tables. This file provides the R code for developing the information placed into the catastrophic health expenditure and impoverishment tables; paper-cheimp.R.
(R) Click here for additional data file.
